# Improving Stem and Progenitor Cell Therapeutics

**DOI:** 10.1155/2016/1409762

**Published:** 2015-12-21

**Authors:** Kenichi Tamama, Kathryn McFadden, Jianjun Guan

**Affiliations:** ^1^Department of Pathology, University of Pittsburgh School of Medicine, Pittsburgh, PA 15261, USA; ^2^McGowan Institute for Regenerative Medicine, University of Pittsburgh, Pittsburgh, PA 15219, USA; ^3^Department of Pathology, The University of Michigan Medical School, Ann Arbor, MI 48109, USA; ^4^Department of Materials Science and Engineering, The Ohio State University, 2041 Collage Road, Columbus, OH 43210, USA

Cell therapy with stem/progenitor cells is a new and promising therapeutic approach for many diseases that currently have few satisfying treatments. The prototype of stem cell-based therapy is bone marrow transplantation, which introduces hematopoietic stem cells (HSCs) to restore hematopoiesis after myeloablation in hematologic malignancy such as leukemia. Besides HSCs, other types of stem/progenitor cells, such as mesenchymal stem cells (MSCs) or induced pluripotent stem cells (iPSCs), are or will be applied in clinical studies.

There are various issues related to stem and progenitor cell therapeutics. First, the therapeutic effects of stem and progenitor cell therapeutics may be inadequate; the beneficial results of cell therapy in initial small-scale clinical studies have not always been reproduced by subsequent large-scale studies. For example, MSCs were shown to be no more effective than placebo in a large-scale, placebo-controlled phase III clinical trial for steroid-resistant graft-versus-host disease (GVHD) [1]. Moreover, autologous bone marrow mononuclear cells did not improve recovery of postmyocardial infarction left ventricular (post-MI LV) function in 2 randomized controlled trials with patients with ST-segment elevation myocardial infarction [2, 3]. These results strongly indicate the urgent needs of further optimization of cell-based therapy.

Safety is a second concern. For example, the original protocol of iPSC generation requires retrovirus-based transduction with 4 transcription factors (c-myc, oct4, klf4, and sos2) or Yamanaka factors [4, 5]. This means that iPSCs generated by the original protocol are potentially subject to insertional mutagenesis, as addressed in a review article by M. G. Cefalo et al. in this special issue. Obviously, there is still great room for improvement in stem/progenitor cell-based therapeutics, and this is the focus of this special issue.

Approaches and strategies to improve the therapeutic potential of stem/progenitor cells include enhancing treatment effect, improving cell delivery to target organs, and promoting cell engraftment and survival after implantation. In their original research article, R.-P. Zhang et al. demonstrated the combination of neurotrophin 3- (NT3-) transduction in a magnetically guided cell targeting system improves the neural regenerative effects of transplanted MSCs in a rat spinal cord injury model. C. Kiratipaiboon et al. showed in their original research article that a quinolone antibiotic ciprofloxacin improves the stemness of human dermal papilla cells by activating Wnt/*β*-catenin signaling, independently of its antimicrobial action. M. Chen et al. showed in their research article that polydactin, a glucoside of resveratrol widely used in traditional Chinese remedies, exerts antioxidative effects by activating the Nrf 2/ARE pathway. Finally, L. Wang et al. demonstrate the efficacy of transplanted umbilical cord mesenchymal stem cells, isolated according to their previously established novel protocol, in reducing standard measures of disease activity in clinical patients with juvenile idiopathic arthritis.

A promising technical approach is 3D-based cell culture. Mammalian cells have been traditionally cultured on the plastic in a 2D condition, but 3D spheroidal aggregates of MSCs or MSC spheroids have been shown to possess enhanced therapeutic potential, as summarized by Z. Cesarz and K. Tamama. In their original research for this special issue, X. Zhao et al. additionally indicate transplanted adipose-derived MSC spheroids exert stronger tissue reparative effects with better graft survival in a rat renal ischemia-reperfusion injury model.

A biomaterial-based approach is another technical advance shown to improve both cell engraftment and survival after transplantation. This is well summarized by X. Li et al. in this issue. The original research by C. Uribe-Cruz et al. more specifically demonstrates whole bone marrow cells encapsulated by alginate-based hydrogel improve survival in rats after subtotal hepatectomy.

In summary, this special issue encompasses both comprehensive reviews and original research highlighting specific approaches, strategies, and techniques designed to improve stem and progenitor cell therapeutics.
